# Longitudinal Study of Changes in Daily and Hourly Steps During the COVID-19 Pandemic in Japan

**DOI:** 10.2188/jea.JE20250328

**Published:** 2026-06-05

**Authors:** Nanae Matsumoto, Masamitsu Kamada, Hana Hayashi, Naoki Kondo, Ichiro Kawachi

**Affiliations:** 1 Department of Health Education and Health Sociology, School of Public Health, Graduate School of Medicine, The University of Tokyo, Tokyo, Japan; 2 Down to Earth, Inc., Chiba, Japan; 3 Keio Global Research Institute, Keio University, Tokyo, Japan; 4 Department of Social Epidemiology and Global Health, Graduate School of Medicine and School of Public Health, Kyoto University, Kyoto, Japan; 5 Department of Social and Behavioral Sciences, Harvard T.H. Chan School of Public Health, Boston, MA USA

**Keywords:** exercise, COVID-19, diurnal variation, epidemiology

## Abstract

**Background:**

The impact of the coronavirus disease 2019 (COVID-19) pandemic on changes in physical activity, particularly diurnal patterns, remains unclear. We investigated temporal changes in daily steps before and after the emergency declaration in Japan during the COVID-19 pandemic.

**Methods:**

Nationwide de-identified data from users of a physical activity-promoting smartphone application were collected. Daily and hourly steps were measured using smartphones from January 2019 to September 2020. Linear mixed models estimated changes in steps before, during, and after the April and May 2020 emergency declaration relative to 2019, involving 3,480 users (daily steps) and 3,402 users (hourly steps).

**Results:**

Compared to the 2019 baseline, daily steps decreased during the emergency declaration (April: −1,115; 95% confidence interval [CI], −1,233 to −998 steps/day) and only partly recovered thereafter (July: −496; 95% CI, −609 to −382 steps/day). This decline was greater among participants aged 18–39 years (*P* for interaction <0.05). By time of day, steps significantly decreased during weekday morning commutes and at night (eg, 21:00: −136; 95% CI, −153 to −119 steps/hour) and during weekend days and late evenings (eg, 12:00: −173; 95% CI, −196 to −151 steps/hour). After the declaration was lifted, step counts recovered but were still lower at night (eg, 21:00 on weekends: −120; 95% CI, −135 to −106 steps/hour).

**Conclusion:**

Daily steps decreased after the emergency COVID-19 declaration in Japan. Even after the emergency period ended, there was a persistent population-level decline in daily steps, with a partial shift in the diurnal pattern. Efforts are needed not only to restore but also to further promote physical activity beyond pre-pandemic levels.

## INTRODUCTION

Physical inactivity is prevalent worldwide, posing a risk of chronic diseases, disorders, and premature mortality.^1^ Mobility restrictions (eg, shelter-in-place orders, curfews, lockdowns) implemented to prevent the spread of the coronavirus disease 2019 (COVID-19) substantially disrupted the daily lives of people worldwide^2^ and resulted in a decrease in physical activity during the early stages of the pandemic.^3^^–^^6^ However, whether cessation of the restrictions returned physical activity to pre-restriction levels remains unclear.^4^^,^^7^^,^^8^

Identifying diurnal patterns in physical activity may inform temporally and demographically targeted interventions, given that activity levels fluctuate throughout the day and differ across population subgroups.^9^^,^^10^ A United States study using large-scale mobile data^11^ revealed that during lockdown, step counts sharply dropped during weekday work commute time (7:00 and 15:00 hours).^11^ This decline in work-related travel was a major contributor to the overall decline in physical activity.^11^ In Japan, the government declared a state of emergency, prompting people to voluntarily restrict their mobility. The impact of this “soft” approach on the diurnal pattern of physical activity remains unclear, particularly at the national level.^8^^,^^12^^,^^13^ To our knowledge, no previous study has investigated this impact throughout the period—before, during, and after the pandemic restrictions—using nationwide data.

Therefore, the aim of this study was to assess temporal changes in physical activity before, during, and after the COVID-19 emergency declaration in Japan in April and May 2020, including an examination of diurnal changes in physical activity.

## METHODS

### Research design and study population

We used data from a smartphone application (app) called “Pa-League Walk,” in which users’ step counts were measured continuously. The app, designed for fans of the Japanese professional Pacific Baseball League, is free and based on gamification principles (see eMaterial 1 and eFigure 1).^14^ The app can be used anywhere and anytime; thus, the users were not limited to stadium visitors. The de-identified data of the app users were provided by Pacific League Marketing, Inc. (Tokyo, Japan).

**Figure 1.**
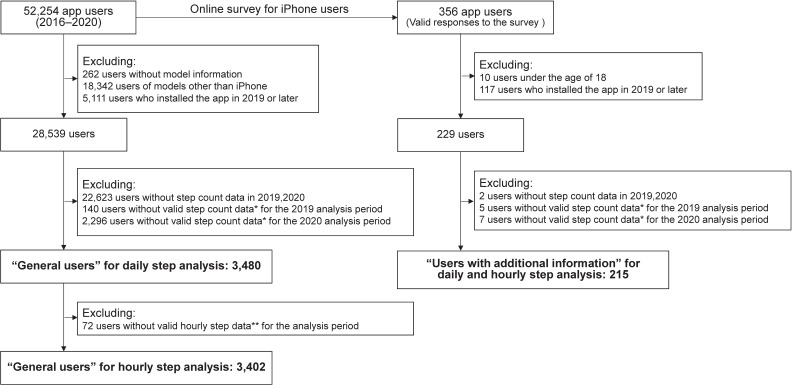
Flowchart of study participants. ^*^Valid step count data are defined as daily step count data that are between 500 steps/day and 100,000 steps/day, and that do not have the same number of consecutive steps as the previous day. ^**^Valid hourly step data refers to data remaining after excluding data for days with a daily total of <500 steps/day or >100,000 steps/day, and where the same step value greater than zero was consecutive for three hours or more.

Figure 1 is a flowchart of the study participants. We analyzed the daily and hourly step count data from January 2019 to September 2020 for general users (primary analysis) and users with additional survey information (a limited sample). The primary outcome was a change in step count, which has been reportedly associated with the risk of developing cardiovascular diseases and mortality.^15^^,^^16^ Of the 52,254 users nationwide (from all 47 prefectures) who installed the app between 2016 and 2020, we included only iPhone data (33,650 users), for which the validity of step counting has been demonstrated in previous studies.^17^^,^^18^ The app automatically recorded the number of steps taken. From the 28,539 users who had the app installed by the end of December 2018, we excluded data for days with <500 steps/day or >100,000 steps/day,^9^ and for days with the same step count as the previous day (possibly due to insufficient carrying time or system bugs). Finally, 3,480 individuals had valid daily step count data for the 2019 and 2020 analysis period. For hourly steps, we also excluded days containing at least 3 consecutive hours of steps of the same value greater than zero, to eliminate potential system bugs. A total of 3,402 people were included for the hourly step count analysis.

To examine the effect modification by sociodemographic and other characteristics, we used additional data from an online survey conducted by Pacific League Marketing, Inc. for iPhone users between September 20 and October 10, 2020. Of the 356 users who consented to the survey and had valid demographic data, 215 users were included, as they met the inclusion criteria: being ≥18 years, having installed the app before 2019, and possessing valid step count data for the analysis periods (all with hourly step data).

All users agreed with the app’s privacy policy, which allowed the use of de-identified data. This study used only de-identified data and was approved by the Ethics Committee of the Graduate School of Medicine at the University of Tokyo.

### COVID-19 pandemic and analysis time frame

During the first wave of the COVID-19 epidemic, a state of emergency was declared in seven prefectures in Japan (including the Greater Tokyo Area), which lasted from April 7, 2020 to May 25, 2020^19^ (see details in eTable 1). We defined January to February 2020 as the period before the declaration, April to May 2020 as the period during the declaration, and July to August 2020 as the period after the declaration was lifted. From July to August 2020 (coinciding with the second wave of the epidemic), although no national emergency was declared, each prefecture adopted its own countermeasures. Considering the seasonality of step counts,^20^ each period (months) in 2020 was compared with the same period in 2019.

### Additional data of the users

To examine effect modification, we used the additional data from the online survey as follows: sex, age, body mass index (BMI; weight [kg]/height^2^ [m^2^]), prefecture of residence, educational attainment (junior high school/high school, vocational school/junior college/technical college, or university [4-year course or higher]), equivalent household income (household income divided by the square root of the number of household members), number of household members, frequency of watching professional baseball games at ballparks in 2019–2020 (0 times/year, <6 times/year, ≥6 times/year, or ≥11 times/year), and the frequency of using the Pa-League Walk app (<1 time/day, 1 time/day, or ≥2 times/day).

### Statistical analysis

#### Daily step analysis

To assess the changes in daily step counts before and after the declaration, we estimated the average change in daily steps for each month in 2020 relative to that in 2019. For the primary analysis, we used a linear mixed model with year (2019 and 2020), month (January to September), and year and month interaction as fixed effects and individuals as a random effect. For users with additional information, we adjusted for the following covariates: sex, age, BMI, educational attainment, equivalent household income, number of household members, area of residence (Tokyo, Kanagawa, Saitama, Chiba, Osaka, Hyogo, and Fukuoka Prefectures, where emergency declarations were issued earlier; vs others), frequency of watching baseball games at ballparks, and frequency of using the app. Additionally, to examine effect modification, we conducted subgroup analyses by these covariates.

#### Hourly step analysis

For the hourly step data, we estimated the diurnal pattern of the average number of steps taken and average change in steps in 2020 relative to that in 2019. We used a linear mixed model with year, time of day (0:00 to 23:00), and the interaction between year and time of day as fixed effects and individuals as a random effect. Since the distribution of steps within a day differs between weekdays and weekends,^9^ we constructed models for weekdays and weekends for each period before, during, and after the declaration. Additionally, to examine effect modification among the 215 participants with additional covariate information, we estimated the amount of change in steps by time of day for each covariate group. We used a linear mixed model adjusting for other covariates.

SAS version 9.4 (SAS Institute Inc., Cary, NC, USA) was used for statistical analysis and Python 3.8 was used for data processing and visualization. The two-tailed significance level was set at 5%. Exploratory comparisons across hourly time points were made without multiple-testing adjustment to illustrate temporal patterns rather than establish definitive significance at each time point.

## RESULTS

The sample dataset contained 1,463,591 person-days of steps from 3,480 users (51.9% female; mean age 41.0; standard deviation [SD], 12.8 years; mean BMI 23.2; SD, 4.5 kg/m^2^: 26.0% overweight [BMI ≥25 kg/m^2^], see eTable 2). The characteristics of the 215 users included in the subgroup analysis are shown in Table 1. Most of the users were aged in their 40s and 50s; 24% of them had a baseline step-count (the average steps in 2019) ≥10,000 steps/day, and less than half of the users had <7,500 steps/day (considered as physically inactive).^21^

**Table 1.** Characteristics of users with additional information for subgroup analysis (2019 and 2020, *n* = 215)

	*n* (%)
Age, years, mean (SD)	45.9 (10.6)
18–29	17 (7.9)
30–39	35 (16.3)
40–49	73 (34.0)
50–59	76 (35.3)
60–67	14 (6.5)
Sex, female	81 (37.7)
BMI, kg/m^2^, mean (SD)	24.0 (4.3)
≥25	78 (36.3)
Educational attainment	
Junior high school or high school	50 (23.3)
Vocational/technical school or junior college	42 (19.5)
4-year college or higher	123 (57.2)
Equivalent household income,^a^ JPY	
≤3,000,000	71 (33.0)
3,000,001–4,999,999	60 (27.9)
≥5,000,000	84 (39.1)
Number of persons in household	
1 person	45 (20.9)
More than 2 people	170 (79.1)
Area of residence	
7 prefectures^b^	139 (64.7)
Other prefectures	76 (35.3)
Frequency of visiting ballparks, times/year	
0	20 (9.3)
1–5	81 (37.7)
6–10	43 (20.0)
≥11	71 (33.0)
Frequency of app use	
Less than once a day	61 (28.4)
Once a day	73 (34.0)
At least twice a day	81 (37.7)
Baseline steps,^c^ steps/day, mean (SD)	8,381 (3,403)
<5,000	25 (11.6)
5,000–7,499	76 (35.3)
7,500–9,999	63 (29.3)
≥10,000	51 (23.7)

BMI, body mass index; SD, standard deviation.

^a^Equivalent household income was calculated by dividing household income by the square root of the number of household members.

^b^Tokyo, Kanagawa, Saitama, Chiba, Osaka, Hyogo, and Fukuoka Prefectures, where emergency declarations were issued on April 7, 2020, prior to the rest of Japan.

^c^The average number of steps for the 2019 step analysis period.

### Daily step analysis

**Figure 2.**
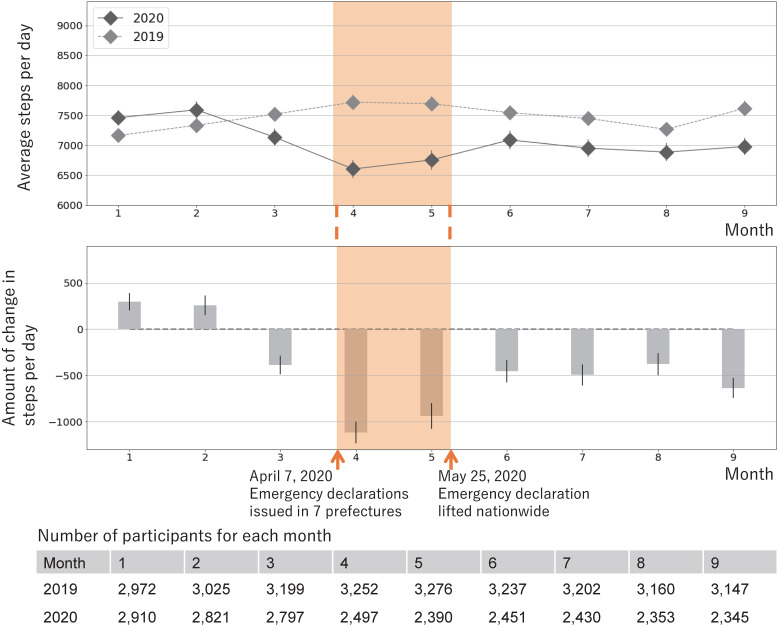
Change in daily steps before, during, and after the declaration of emergency in Japan (2019 and 2020, *n* = 3,480). The average number of steps in January–September 2019 and 2020 (top row) and the average change in steps in 2020 relative to 2019 for each month (bottom row) were estimated using a linear mixed model. Error bars indicate 95% confidence intervals. A negative value of change means the average steps decreased from 2019 to 2020.

Figure 2 shows the average daily steps in each month of 2019 and 2020 as well as the average change in steps from 2019 to 2020. Daily steps in 2019 hovered around 7,500 steps, with seasonal variations (January: 7,162; 95% confidence interval [CI], 7,045–7,274 steps/day], April: 7,719; 95% CI, 7,596–7,842 steps/day). Daily steps in 2020, relative to the same months in 2019, increased slightly in January and February (January: +297; 95% CI, 205–390 steps/day) but decreased from March onwards, with a substantial decrease to −1,115 (95% CI, −1,233 to −998) steps/day in April, when emergency was declared. Although the trend recovered after June 2020, when the declaration was lifted, it remained lower than that in 2019 (July: −496; 95% CI, −609 to −382 steps/day). The same trend was confirmed for users with additional information, despite the small sample size resulting in larger confidence intervals (April: −774; 95% CI, −1,177 to −371 steps/day, July: −122; 95% CI, −582 to 338 steps/day; see eFigure 2).

Subgroup analysis confirmed the effect modification by age, area of residence, frequency of app use, and baseline step count (*P* for interaction <0.05; Table 2). Younger users, those in the seven prefectures where the state of emergency was declared before the rest of the country, those who used the app infrequently, and those with higher baseline step counts showed a greater decrease in daily steps during the declaration. The downward trend in steps continued even after the declaration was lifted. Additionally, although no significant effect modification was observed, users with higher education and income exhibited a larger decrease in steps.

**Table 2.** Change in daily steps before, during, and after the declaration of emergency in subgroups (*n* = 215)

Subgroups	*n*	Change in steps from 2019 to 2020 (95% CI)^a^			*P* for interaction

		January–February (Before DoE)	April–May (During DoE)	July–August (After DoE)	
Age, years					**0.001**
18–39	52	80 (−326 to 485)	−2,203 (−2,857 to −1,549)	−1,006 (−1,590 to −423)	
40–67	163	481 (151 to 812)	−463 (−890 to −36)	219 (−325 to 762)	
Sex					0.294
Male	134	416 (48 to 783)	−752 (−1,259 to −245)	−75 (−550 to 401)	
Female	81	373 (−22 to 768)	−832 (−1,330 to −334)	64 (−898 to 1,026)	
BMI, kg/m^2^					0.383
<25	137	512 (160 to 864)	−656 (−1,116 to −196)	−137 (−582 to 308)	
≧25	78	229 (−216 to 674)	−967 (−1,612 to −323)	147 (−822 to 1,115)	
Education (bachelor’s degree)					0.058
No	92	398 (20 to 776)	−438 (−988 to 113)	255 (−564 to 1,074)	
Yes	123	411 (18 to 804)	−1,045 (−1,556 to −534)	−252 (−747 to 243)	
Income^b^, JPY					0.069
≦4,020,000	101	424 (−34 to 882)	−216 (−812 to 379)	234 (−293 to 760)	
>4,020,000	114	385 (48 to 723)	−1,248 (−1,709 to −787)	−264 (−975 to 447)	
Household size					0.177
1 person	45	930 (386 to 1,474)	−673 (−1,628 to 283)	60 (−829 to 948)	
More than 2 people	170	282 (−33 to 596)	−805 (−1,213 to −396)	−56 (−577 to 465)	
Area of residence					**0.001**
7 prefectures^c^	139	373 (43 to 702)	−1,320 (−1,796 to −843)	−524 (−914 to −135)	
Other	76	465 (−37 to 967)	120 (−455 to 695)	828 (−184 to 1,840)	
Visiting ballparks, times/year					0.377
≦5	101	109 (−296 to 513)	−1,024 (−1,557 to −491)	−208 (−694 to 279)	
>5	114	644 (261 to 1,028)	−554 (−1,107 to −1)	111 (−634 to 857)	
Frequency of app use, times/day					**0.002**
≦1	134	243 (−75 to 561)	−1,340 (−1,823 to −857)	−646 (−1,074 to −218)	
>1	81	602 (103 to 1,102)	−87 (−682 to 508)	775 (−85 to 1,636)	
Baseline steps^d^, steps/day					**0.006**
<7,500	101	329 (−76 to 73)	−190 (−761 to 380)	733 (−100 to 1,566)	
≧7,500	114	449 (70 to 828)	−1,227 (−1,712 to −742)	−625 (−1,073 to −176)	

Total	215	405 (128 to 682)	−777 (−1,155 to −400)	−33 (−487 to 420)	

BMI, body mass index; CI, confidence interval; DoE, declaration of emergency.

Bold: *P* for interaction <0.05.

^a^In each subgroup, a linear mixed model was used to estimate the change in daily steps before (January–February), during (April–May), and after (July–August) the declaration of the state of emergency, adjusting for age, sex, BMI, educational attainment, equivalent household income, number of household members, area of residence, frequency of visiting ballparks, and frequency of app use.

^b^Equivalent household income was calculated by dividing household income by the square root of the number of household members.

^c^Tokyo, Kanagawa, Saitama, Chiba, Osaka, Hyogo, and Fukuoka prefectures, where emergency declarations were issued on April 7, 2020, prior to the rest of Japan.

^d^The average number of steps for the 2019 step analysis period.

### Hourly step analysis

**Figure 3.**
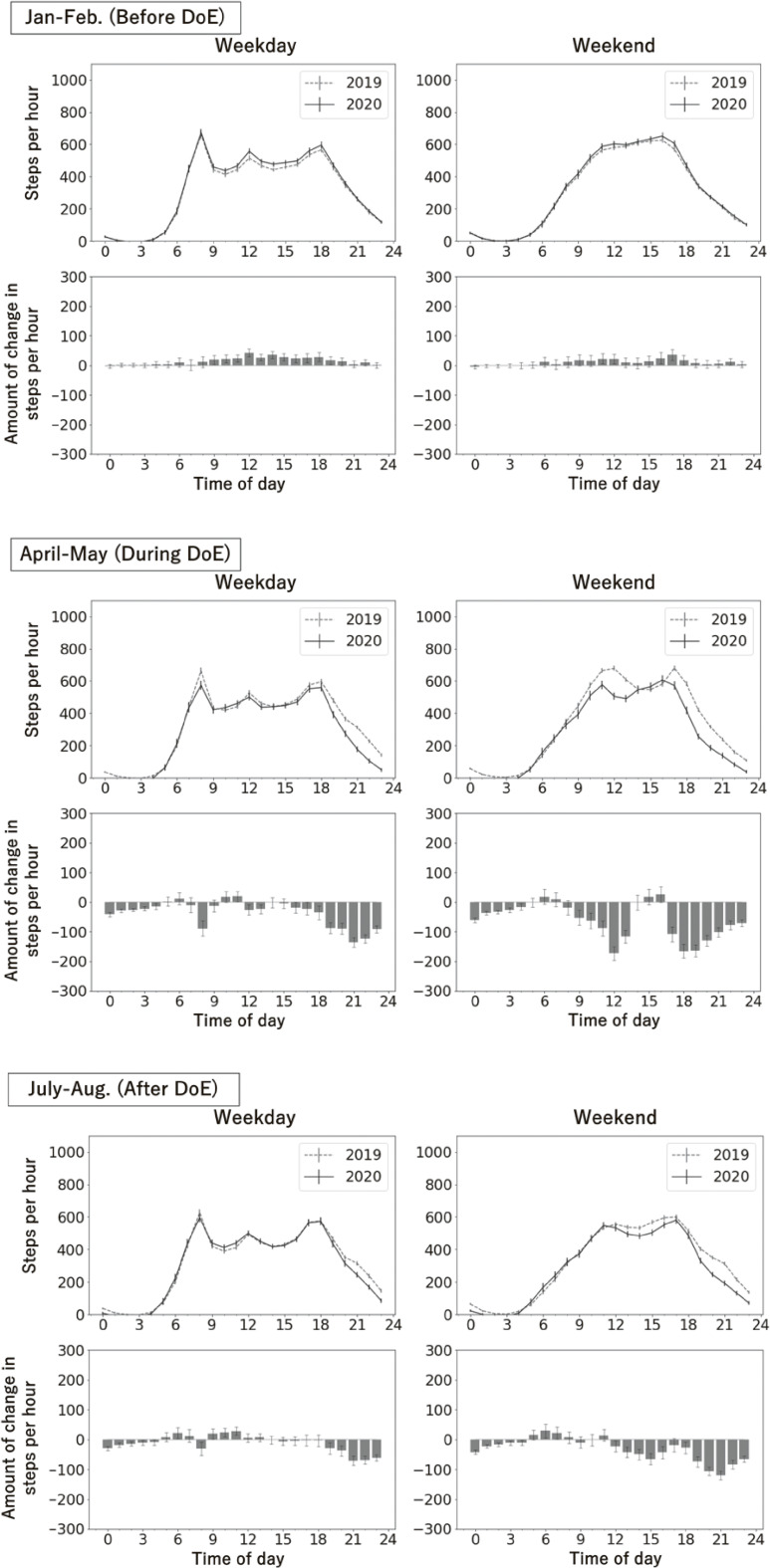
Change in steps taken by time of day before, during, and after the declaration (2019 and 2020, *n* = 3,402). DoE, declaration of emergency. The average number of steps by time of day in 2019 and 2020 (upper panels) and the average change in the number of steps by time of day in 2020 relative to 2019 (lower panels) are shown. Steps were estimated using a general linear mixed model for weekdays (left panels) and weekends (right panels) in the periods before (January–February), during (April–May), and after (July–August) the declaration of the state of emergency. Error bars indicate 95% confidence intervals. A negative value of change means the average steps decreased from 2019 to 2020.

Figure 3 shows the diurnal pattern of average step counts in 2019 and 2020, and the estimated change in steps from 2019 to 2020 for the periods before, during, and after the declaration, separately for weekdays and weekends. The distribution of hourly steps on weekdays had three peaks. The distribution on weekends was unimodal with a peak in the afternoon before the declaration, and bimodal with two peaks in the morning and afternoon during and after the declaration. As for the change in hourly steps from 2019 to 2020, marked decreases were found at 8:00 and evening hours after 18:00 on weekdays during the declaration (−136; 95% CI, −153 to −119 steps/hour at 21:00). On weekends, a marked decrease was observed during the daytime hours of 9:00–14:00 and the evening hours after 17:00 (−173; 95% CI, −196 to −151 steps/hour at 12:00, −165; 95% CI, −184 to −145 steps/hour at 19:00). After the declaration, the decrease in steps on weekdays somewhat recovered during the day (−31; 95% CI, −53 to −9 steps/hour at 8:00, and −71; 95% CI, −86 to −56 steps/hour at 21:00). Steps also recovered on weekends, but a significant decrease was still present in the late evening hours (−120; 95% CI, −135 to −106 steps/hour at 21:00). Similar results were observed for users with additional information (see eFigure 3).

In the subgroup analysis, most subgroups showed a decreasing trend during the declaration in the hours of 8:00 and 18:00, and later in the evening on weekdays (see eFigure 4), and during the hours of 12:00 and 17:00 and later in the evening on weekends (see eFigure 5). Among younger adults, steps decreased at all times during the day after 7:00 on weekdays and after 8:00 on weekends (−128; 95% CI, −204 to −52 steps/hour at 14:00 on weekdays and −315; 95% CI, −451 to −178 steps/hour at 19:00 on weekends). After the declaration was lifted, the decreasing trend continued, particularly on weekends. Conversely, for those with lower income and those living in prefectures with the later declaration, little decrease was observed in steps at 8:00 on weekdays (lower-income group: −3; 95% CI, −126 to 120 steps/hour).

## DISCUSSION

This nationwide longitudinal study demonstrated changes in physical activity levels before and after the emergency declaration in April and May 2020 in Japan, as well as changes in diurnal patterns of physical activity. A marked decrease of approximately 1,000 steps/day was observed during the emergency declaration, which may have clinical significance if persisted.^22^ Although step count activity recovered after the declaration was lifted, daily step counts were lower than those in 2019. This downward trend was pronounced in younger age groups, the seven prefectures where the emergency declaration was issued before the rest of the country, infrequent app users, and those with high baseline step counts. The hourly step analysis during the declaration period showed a significant decrease in step counts in the morning and evening commute times on weekdays and most of the day (except for the early morning and some evening hours) on weekends. After the declaration was lifted, the daytime step counts improved but those of the late evening hours remained low.

The downward trend in daily steps, which began in March before the declaration, was consistent with the findings of previous studies conducted during the early stages of the epidemic in Japan.^6^^,^^8^^,^^13^ In terms of magnitude, the decline in step count during the pandemic was less in Japan than in other countries,^6^^,^^13^ a trend confirmed in this study. Specifically, there was an average decrease of 1,432 steps/day (27.3%) across 187 countries from the World Health Organization’s pandemic declaration on March 11, 2020, to 1 month later in,^6^ while the maximum average decrease in the present study was 987 steps/day (13.0%).

Our study showed that younger users and those with more baseline steps had a larger decline in their daily step counts during and after the declaration. This is consistent with a United Kingdom study^4^ on smartphone app users aged 14–93 years, which reported a 37% reduction in weekly activity, with younger adults experiencing the greatest decline. Furthermore, a study of 1,167 Japanese adults using accelerometers (worn on the wrist, chest, or waist) reported a significant step count decrease in the Tokyo metropolitan area, with reductions of up to 1,800 steps/day.^8^ This was consistent with our study, which showed a more pronounced decrease in step counts in the seven prefectures where the state of emergency was first declared. However, the results of the difference in physical activity change by socioeconomic status differed from those of previous studies.^7^^,^^11^^,^^23^^,^^24^ During the pandemic, physical activity decreased more among people with lower levels of education and income in Western countries.^7^^,^^11^^,^^23^^,^^24^ However, our study showed (albeit non-significantly) that groups with higher education and income had a larger decrease in steps after the emergency declaration, and the decrease tended to continue even after the declaration was lifted. The discrepancy in results could be due to differences in alternative physical activities performed during the pandemic by those with higher incomes and education in foreign countries compared with those in Japan. Higher socioeconomic status linked to increased behavior restriction, including going out for shopping, eating out, and events, alongside telework (or remote learning), seen globally.^25^^–^^27^ Meanwhile, those with lower socioeconomic status might be less likely to lose transport-related and occupational physical activity because of the need to commute to essential work. In Western countries, additional physical activities, such as jogging around the house, compensated for reduced daily mobility.^11^^,^^28^ However, in Japan, such compensatory behaviors might not have been sufficiently adopted or might have been limited to strength training at home, which is not reflected in the step count.^29^ Furthermore, the characteristics of the app may have affected our observed results. A quasi-experimental study showed that the Pa-League Walk app increased physical activity even among people with lower socioeconomic status and that it also successfully involved those who were in the pre-contemplation stage of physical activity behavior change.^14^ The overall average of the decrease in daily steps in this study was less than that in Japanese users of a general health app.^6^ However, the participants in the subgroup analysis were more highly educated than the general population in Japan, and we might have not adequately captured the phenomena that occurred in groups with lower socioeconomic status, including those whose work and income were significantly affected by the pandemic.

Our hourly step analysis showed that during the declaration, steps decreased during the morning and evening hours on weekdays and day and evening on weekends. A United States study using mobile GPS data showed weekday drops around 7:00 and 15:00 during the lockdown, with similar unimodal patterns across weekdays and weekends.^11^ In Japan, physical activity associated with leisure activities on weekends and at night decreased in accordance with government’s request to reduce the opening hours of restaurants, especially bars, clubs, and establishments serving alcoholic beverages,^30^ encouraging voluntary restraint in going out to downtown areas at night,^19^ and the closure of leisure, commercial, and exercise facilities during the emergency. In a study of full-time workers in the Tokyo metropolitan area, a significant decrease in physical activity during nighttime was reported post-emergency using hip-worn accelerometers.^12^ This finding is similar to that of the present study. However, that study did not examine the period during the emergency declaration. The decrease in steps on weekday mornings was less among those in rural areas and those with lower incomes, suggesting differences in teleworking implementation rates between rural and urban areas.^31^ Additionally, steps decreased across all times (on weekdays and weekends) among younger adults, and this downward trend continued even after the emergency was lifted. Although the exact mechanism underlying these age-related differences remains unclear, an early adaptation to telework, online learning, remote entertainment, and socializing among younger generations^32^ may have led to major lifestyle changes related to physical activity. Although the study period ended in September 2020, the decrease in step counts has possibly persisted for a longer time.^6^^,^^8^^,^^12^ Countermeasures against physical inactivity are necessary, considering that changes and diversity in situations and time periods tend to create different opportunities for physical activity. These temporal patterns of physical activity may help inform time-targeted behavioral interventions and public messaging strategies, particularly during periods of restricted mobility.

The strengths of this study included the objective measurement of step count data using a smartphone via a built-in accelerometer, eliminating recall bias. Additionally, we longitudinally analyzed high-density (ie, hourly) data collected throughout Japan. Furthermore, we examined the effect modification of several less investigated factors that could contribute to physical inactivity in Japan.^6^^,^^8^^,^^13^

However, this study had some limitations. First, we did not collect information on the time spent carrying smartphones. Hence, we could not consider the amount of physical activity performed when smartphones were not being carried. In Japan, the time spent at home increased during the declaration,^33^ during which if smartphones were being carried less, the decrease in physical activity may have been overestimated.^17^ In an attempt to get around this, we excluded data with <500 steps/day. Second, we did not consider the effects of meteorological factors, such as precipitation and outdoor temperature.^34^^,^^35^ For example, in January, the average daily steps increased by 297 steps/day from 2019 to 2020, coinciding with a warmer winter when the national average temperature was 2.0°C higher in 2020.^36^ Turning to summer as our primary hypothesis testing period, the average temperature in Tokyo from July to September was 25.9°C in both 2019 and 2020, and precipitation was slightly higher in 2019 (500.0 mm) than in 2020 (449.5 mm).^37^ Therefore, the observed decrease in steps in 2020 is unlikely to be attributed to meteorological factors. Last, we analyzed data from users of an app designed to promote physical activity among baseball fans in Japan, which limits the generalizability of our findings. Users of the app may differ from the general population in terms of demographic and lifestyle factors, such as higher baseline physical activity and greater health awareness. These characteristics could lead to an underestimation of pandemic-related declines in physical activity and may limit the applicability of our findings to less active or more vulnerable populations. Moreover, these results could include changes in physical activity associated with the continued use of the app or lifestyle changes during the pandemic. Particularly, in additional analysis, users demonstrated higher app engagement and baseline physical activity. The suspension of baseball games may have also led to a decrease in steps among enthusiastic fans. Nonetheless, considering that the changes in physical activity over time and the impact of effect modifiers on physical activity during the pandemic have not been previously investigated, we believe that this study would help clarify the influence of the pandemic on physical activity. Furthermore, it can potentially guide in planning countermeasures against physical inactivity.

### Conclusions

This nationwide longitudinal study revealed a substantial decline in step count in Japan during the COVID-19 emergency declaration, with pronounced effects in younger populations. Hourly step data demonstrated a significant decrease in physical activity during weekday commute times and across most of the weekend. Although the step count recovered post-declaration, the overall step count remained below 2019 levels (especially during late evenings). As the impact of the pandemic has been prolonged^6^ and many countries, including Japan, continue to experience a persistent decline in physical activity,^6^^,^^38^ implementing countermeasures against physical inactivity are urgently needed. Future strategies should incorporate diurnal activity patterns while also addressing broader individual and environmental factors that influence physical activity.

## SUPPLEMENTARY MATERIAL

eMaterial 1. Pa-League Walk app features

eTable 1. COVID-19 related events in Japan (January to September 2020)

eTable 2. Age and sex distribution of general users (2019 and 2020; n = 3,480)

eFigure 1. Screenshot of the app “Pa-League Walk” (© Pacific League Marketing, Inc.).

eFigure 2. Change in daily steps for users with additional information before, during, and after the declaration of emergency in Japan (2019 and 2020, n = 215).

eFigure 3. Change in steps by time of day before, during, and after the declaration of an emergency among users with additional information (2019 and 2020, n = 215).

eFigure 4. Change in steps by time of day on weekdays before, during, and after the declaration of emergency in subgroups (weekday in 2019 and 2020, n = 215).

eFigure 5. Change in number of steps by time of day before, during, and after emergency declaration in subgroups (weekends in 2019 and 2020, n = 215).
