# Supplementary material for: Longitudinal Study of Changes in Daily and Hourly Steps During the COVID-19 Pandemic in Japan
Source: J Epidemiol. 2026 Jun 5;36(6):179–87. doi: 10.2188/jea.JE20250328 (PMC13158354; doi:10.2188/jea.JE20250328)
Supplement: Supplementary file 1 [file je-36-179-s001.pdf]

Supplementary Materials

eMaterial 1. Pa-League Walk app features

The Pa-League Walk app includes features such as a function that allows fans of opposing teams to compete against each other for the total number of steps taken during a game day. In addition, users receive a digital photo of a player from the team they support if they walk ≥10,000 steps in a day (see eFigure 1).

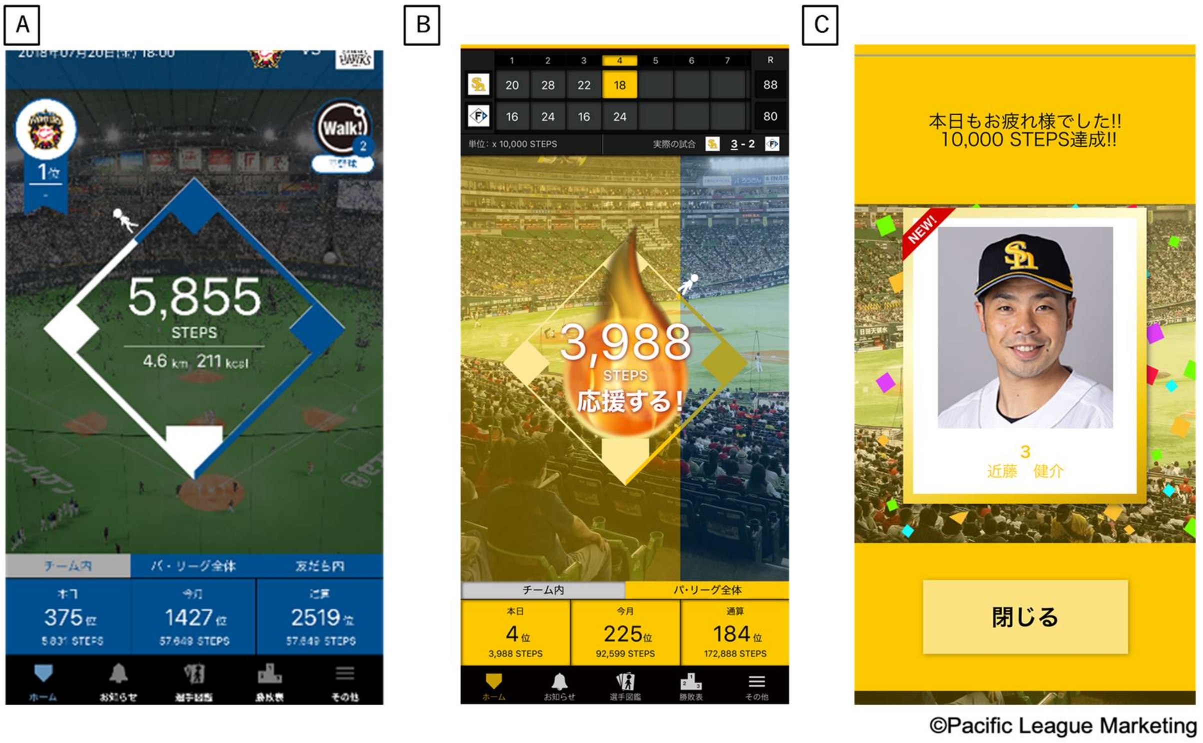

eFigure 1. Screenshot of the app "Pa-League Walk" (© Pacific League Marketing, Inc.)

Screen design themed around the team colors of the team you support: (**A**) home screen, (**B**) walk-off mode during actual professional baseball game time, and (**C**) digital photos of the players you get when you reach 10,000 steps per day.

eTable 1. COVID-19-related events in Japan (January to September 2020)

Date	Events
1/15/2020	First positive case confirmed in Japan
2/3/2020	Cruise ship with infected passengers enters port of Yokohama
2/13/2020	First confirmed death of a positive case in Japan.
3/2/2020	Request for temporary closure of elementary and junior high schools nationwide
3/9/2020	Decision to postpone the start of the professional baseball season
3/11/2020	World Health Organization declares COVID-19 pandemic
4/7/2020	A state of emergency declared in seven prefectures.*
4/11/2020	A record number of 720 positive cases per day in Japan (peak of the first wave)
4/16/2020	Emergency declaration extended nationwide
5/7/2020	Daily number of positive cases in Japan drops below 100
5/14/2020	State of emergency declaration lifted in 39 prefectures
5/25/2020	State of emergency declaration lifted nationwide
6/1/2020	Temporary school closures end at elementary and junior high schools nationwide
6/19/2020	Nationwide easing of requests for voluntary restraint in cross-prefecture travel.
6/19/2020	Professional baseball league opens without spectators
7/3/2020	Daily positive cases in Japan exceed 200 for the first time in two months.
7/10/2020	The maximum number of people allowed to hold a large-scale event raised from 1,000 to 5,000, and professional baseball leagues also began admitting spectators up to this limit.
7/22/2020	Record 795 positive cases in Japan in one day
7/22/2020	Launch of the "Go To Travel" Campaign, a Japanese government subsidy encouraging domestic travel to help boost the economy.
7/22/2020	Decision to postpone the relaxation of the maximum number of people allowed to hold a large-scale event until the end of August.
7/29/2020	More than 1,000 positive cases per day in Japan
8/7/2020	A record number of 1,605 positive cases per day in Japan (second wave peak)**
9/19/2020	Relaxation of attendance limits for a large-scale event was followed by an increase in the spectator cap for professional baseball leagues to 50% of stadium capacity.

COVID-19, coronavirus disease 2019.

*Unlike mandatory lockdowns enforced in some countries, the state of emergency in Japan had few legally binding restrictions and imposed only minimal limitations on private rights, relying primarily on voluntary compliance. Prefectural governors requested residents to cooperate as necessary to prevent infection, for example, by refraining from going out except when necessary to maintain their daily lives. They also demanded the closure of schools and restrictions on the use of facilities that attract an unspecified number of people such as department stores and movie theaters.

**During the second wave of the COVID-19 epidemic, although no emergency declaration was issued, each prefecture took its own measures to deal with COVID-19. For example, in Tokyo, people were asked to refrain from going out at night to downtown areas, and restaurants and karaoke bars that served alcoholic beverages were asked to shorten their business hours to 22:00 from August 3, 2020.

References

1. Cabinet Secretariat, Summary of the Emergency Declaration on Countermeasures for Novel Coronavirus Infections.
   https://corona.go.jp/news/news_20200421_70.html
2. Cabinet Secretariat, Countermeasures for Novel Coronavirus Infections, Countermeasures Headquarters and other information.
   https://corona.go.jp/expert-meeting/

3. NHK, Special Service, Novel Coronavirus Infections, Full Chronology of the COVID-19 related articles. https://www3.nhk.or.jp/news/special/coronavirus/chronology/?mode=all&target=202006
4. NHK, Special section on the number of domestic infections with the Novel Coronavirus. https://www3.nhk.or.jp/news/special/coronavirus/data-all/
5. Ministry of Education, Culture, Sports, Science and Technology, Status of Temporary Closure of Schools in Response to the Novel Coronavirus Infection. https://www.mext.go.jp/content/20200513-mxt_kouhou02-000006590_2.pdf
6. The Director of the Office for COVID-19 and Other Emerging Infectious Disease Control, Cabinet Secretariat, Restrictions on holding events until the end of November. https://corona.go.jp/news/pdf/jimurenraku_20200911.pdf
7. Tokyo Metropolitan Government, The 35th Tokyo Metropolitan Government COVID-19 Task Force Meeting Materials (July 30, 2020). https://www.bousai.metro.tokyo.lg.jp/taisaku/saigai/1007288/1009999.html

eTable 2. Age and sex distribution of general users (2019 and 2020; n=3,480)

	Total n=3,480	Male n=1,603	Female n=1,806
Age, years, mean (SD)	41.0 (12.8)	43.7 (12.1)	38.6 (12.9)
18−29	643 (18.5)	155 (9.7)	487 (27.0)
30−39	802 (23.0)	342 (21.3)	458 (25.4)
40−49	981 (28.2)	559 (34.9)	420 (23.3)
50−59	770 (22.1)	421 (26.3)	346 (19.2)
60−67	168 (4.8)	90 (5.6)	78 (4.3)
Other/Missing	116 (3.3)	36 (2.2)	17 (0.9)

SD, standard deviation.

Figures are n (%) unless stated otherwise. Data from individuals with missing sex information are excluded from the sex-based breakdown (n=71, 2.0%).

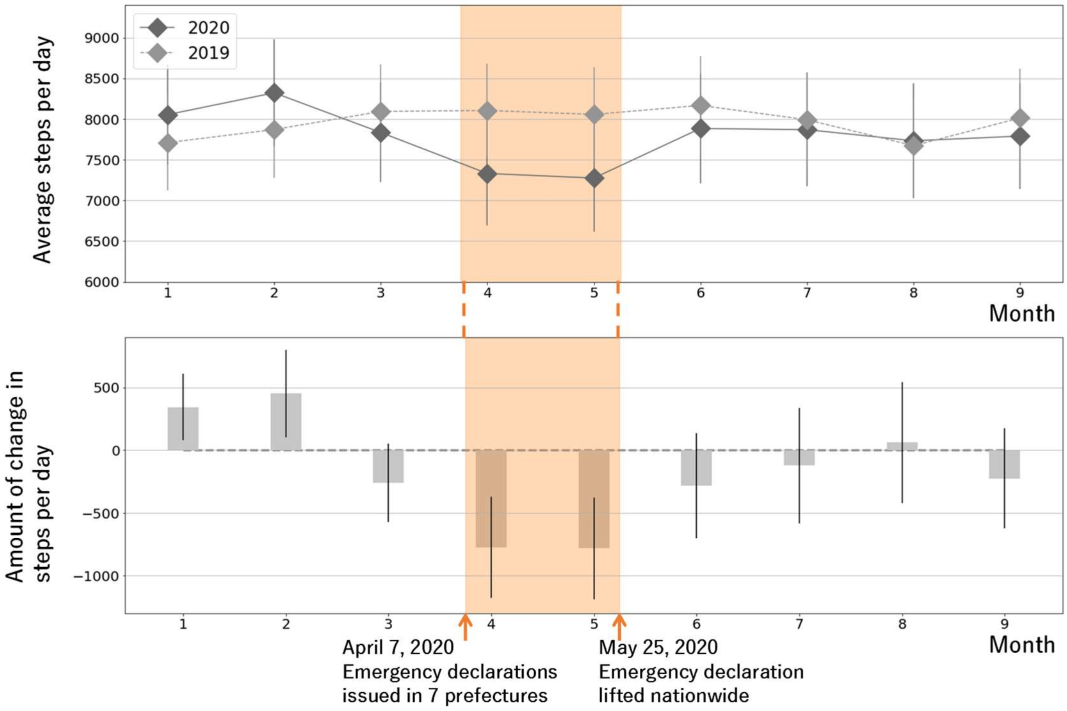

Number of participants for each month

Month	1	2	3	4	5	6	7	8	9
2019	208	209	209	211	210	213	210	211	213
2020	198	201	205	199	194	200	204	204	210

eFigure 2. Change in daily steps for users with additional information before, during, and after the declaration of emergency in Japan (2019 and 2020, n=215)

The figure shows the average number of steps in January−September 2019 and 2020 (top row) and the average change in steps in 2020 relative to 2019 for each month (bottom row), estimated using a linear mixed model and adjusting for age, gender, body mass index, education, income, household size, region of residence, frequency of watching ballparks, and frequency of app use. Error bars indicate 95% confidence intervals. A negative value of change means the average steps decreased from 2019 to 2020.

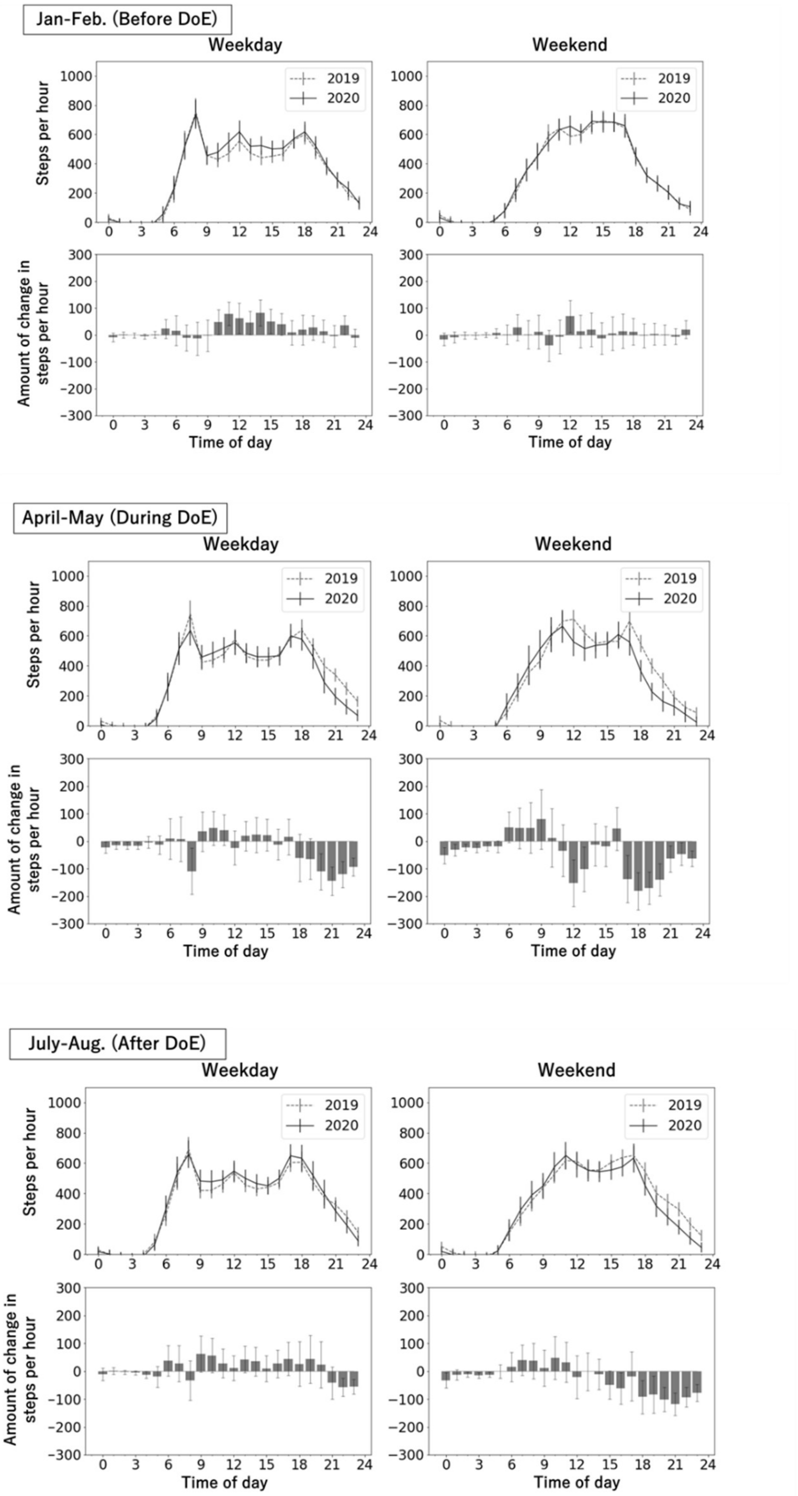

eFigure 3. Change in steps by time of day before, during, and after the declaration of emergency among users with additional information (2019 and 2020; n=215)

DoE, declaration of emergency. The average number of steps by time of day in 2019 and 2020 (upper panels) and the average change in number of steps by time of day in 2020 relative to 2019 (lower panels) are shown. Steps were estimated with a general linear mixed model for weekdays (left panels) and weekends (right panels) in the periods before (January−February), during (April−May), and after (July−August) the declaration of a state of emergency, adjusting for age, gender, BMI, education, income, household size, region of residence, frequency of watching ballparks, and frequency of app use. Error bars indicate 95% confidence intervals. A negative value of change means the average steps decreased from 2019 to 2020.

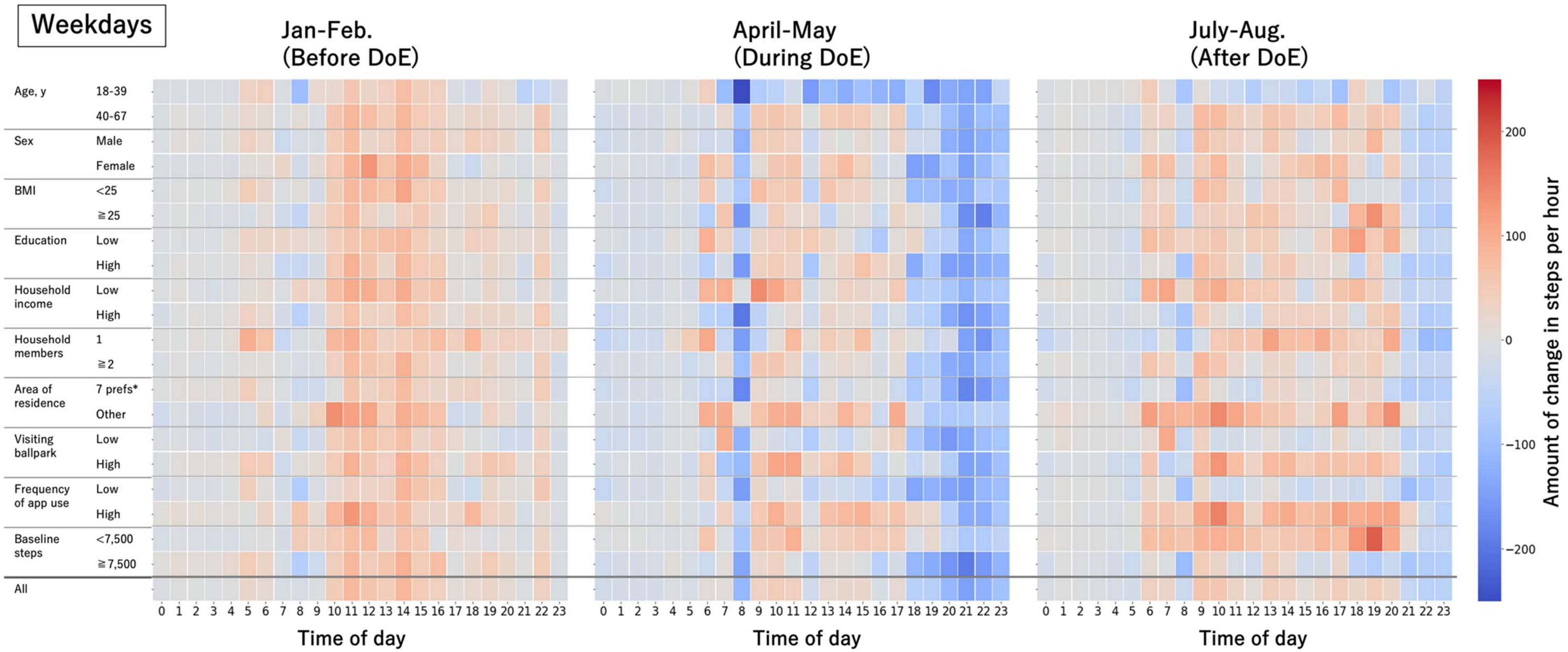

eFigure 4. Change in steps by time of day on weekdays before, during, and after the declaration of emergency in subgroups (weekdays in 2019 and 2020, n=215)

DoE, declaration of emergency; BMI, body mass index. The heatmap shows the average change in steps by time of day from 2019 to 2020 for weekdays in each of the periods before (January−February), during (April−May), and after (July−August) the declaration of emergency, estimated for each subgroup using linear mixed models, adjusting for age, gender, BMI, education, income, household size, area of residence, frequency of watching ballparks, and frequency of app use. The warm (cold) color of the graph indicates an increase (decrease) in the number of steps from 2019 to 2020, with darker colors indicating larger amounts of change.
*Tokyo, Kanagawa, Saitama, Chiba, Osaka, Hyogo, and Fukuoka prefectures, where the declaration of emergency was issued on April 7, 2020, prior to the rest of Japan.

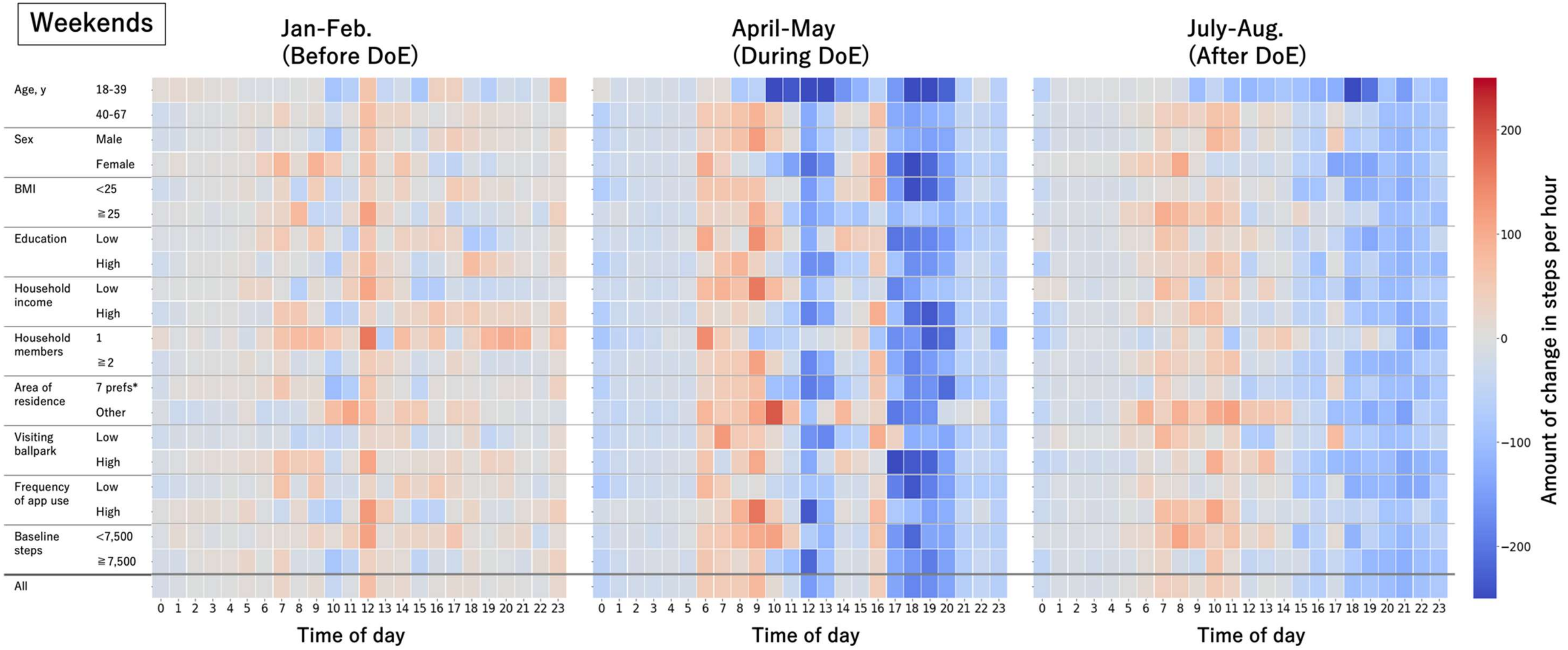

eFigure 5. Change in number of steps by time of day before, during, and after emergency declaration in subgroups (weekends in 2019 and 2020, n=215)

DoE, declaration of emergency. The following table shows the average change in the number of steps by time of day for weekends in each of the periods before (January−February), during (April−May), and after (July−August) the declaration of a state of emergency, estimated for each attribute group using linear mixed models, adjusting for age, gender, BMI, last education, equivalent household income, number of household members, region of residence, frequency of watching ballparks, and frequency of launching apps, relative to 2019 The warm colors in the graph indicate an increase in the number of steps. The warm color of the graph indicates an increase in the number of steps, and the cold color indicates a decrease in the number of steps, with darker colors indicating larger absolute values.
*Tokyo, Kanagawa, Saitama, Chiba, Osaka, Hyogo, and Fukuoka prefectures, where the declaration of emergency was issued on April 7, 2020, prior to the rest of Japan.
